# Transcriptomic data on the role of PEST-domain-enriched tyrosine phosphatase in the regulation of antigen-mediated activation and antiallergic action of glucocorticoids in mast cells

**DOI:** 10.1016/j.dib.2018.08.188

**Published:** 2018-09-05

**Authors:** George K. Ainooson, Victor Gourain, Michael Stassen, Andrew C.B. Cato

**Affiliations:** a Karlsruhe Institute of Technology, Institute of Toxicology and Genetics, Hermann-von-Helmholtz-Platz-1, 76344 Eggenstein-Leopoldshafen, Germany; b Institute for Immunology, University Medical Center, Johannes Gutenberg University, Langenbeckstr. 1, 55131 Mainz, Germany

**Keywords:** BMMCs, Bone marrow derived mast cells, COX-2, Cyclooxygenase 2, CSF2, Colony stimulating factor 2, DAVID, Database for Annotation, Visualization and Integrated Discovery, DEX, Dexamethasone, DNP, Dinitrophenol, IgE, Immunoglobulin E, IL 13, Interleukin 13, IMDM, Iscove׳s Modified Dulbecco׳s Medium, KEGG, Kyoto Encyclopedia of Genes and Genomes, PCA, Principal Component Analysis, PEP, PEST-domain-enriched tyrosine phosphatase, PTGDS, Prostaglandin D2 synthase - lipocalin type, qRT-PCR, Quantitative real-time polymerase chain reaction, RNA-seq, RNA-sequencing, SBS, Sequencing by Synthesis, TNFα, Tumor necrosis factor alpha, RNA sequencing, Quantitative real-time PCR, Allergy, Gene expression, Phosphatase

## Abstract

Protein tyrosine phosphatases and glucocorticoids are known to regulate allergic and antiallergic action in activated mast cells. Here we provide RNA sequencing and quantitative real-time PCR data from bone marrow derived mast cells, for wild-type and PEST-domain-enriched tyrosine phosphatase (PEP) null mice, activated by immunoglobulin E sensitization and dinitrophenol treatment, and additionally treated with the glucocorticoid dexamethasone. The transcriptomics experiment was performed in duplicate with a total of 16 samples (GSE108972).

**Specifications table**

**Table:** 

Subject area	*Immunology*
More specific subject area	*Allergy*
Type of data	*Transcriptome, figures, tables*
How data was acquired	*High-throughput RNA sequencing using Illumina HiSeq. 1500, quantitative real-time PCR (qRT-PCR) using SYBR Green dye in a StepOnePlus™ Real-Time PCR system*
Data format	*Processed*
Experimental factors	*Wild-type versus mutant cells*
Experimental features	*Comparison of mRNA from PEP+/+ and PEP−/− mouse bone marrow derived mast cells (BMMCs) following activation with the antigen dinitrophenol and additional treatment with the glucocorticoid Dexamethasone.*
Data source location	*Karlsruhe Institute of Technology, Institute of Toxicology and Genetics Hermann-von-Helmholtz-Platz-1, 76344 Eggenstein-Leopoldshafen, Germany*
Data accessibility	http://www.ncbi.nlm.nih.gov/geo/query/acc.cgi?acc=GSE108972

**Value of the data**

• This dataset is a transcriptomic analysis of bone marrow derived mast cells (BMMCs) from PEP+/+ and PEP−/− mice activated by antigen and additionally treated with glucocorticoids.
• These data provide RNA-seq and qRT-PCR gene expression results to show the role of PEP in antigen-mediated mast cell activation and the inhibition of mast cell activation by glucocorticoids.
• These data will serve as a reference point for antigen-mediated activation of mast cells and the antiallergic action of glucocorticoids.

## 1 Data

The data show gene expression profiles of 16 RNA-sequencing (RNA-seq) samples, representing two replicates each from BMMCs isolated from wild-type (PEP+/+ ) and mutant (PEP−/−) mice:

• sensitized with Immunoglobulin E (IgE) (500 ng/ml) for 3 h
• sensitized with IgE (500 ng/ml) for 3 h and activated with the antigen dinitrophenol (DNP) (as dinitrophenyl -bovine serum albumin, 200 ng/ml) for 1 h
• treated with dexamethasone (DEX) (10^−7^ M) for 8 h and IgE (500 ng/ml) for 3 h
• treated with DEX (10^−7^ M) for 8 h, IgE (500 ng/ml) for 3 h and DNP (200 ng/ml) for 1 h.

The data are deposited under the Gene Expression Omnibus (GEO) number GEO: GSE 108972 at http://www.ncbi.nlm.nih.gov/geo/query/acc.cgi?acc=GSE108972.

## 2 Experimental design, materials and methods

### 2.1 Preparation of the biological material

BMMCs from PEP+/+ and PEP−/− mice were isolated by culturing bone marrow cells from the femur and tibia of male C57BL/6 mice aged 8–10 weeks in mast cell medium (IMDM supplemented with 10% foetal calf serum, 2 mM L-glutamine, 1 mM pyruvate, 100 ng stem cell factor/kit ligand, 5 ng/ml IL3, 50 µM β-mercaptoethanol, 1% penicillin/streptomycin 10,000 U/ml) in T-75 flask at 37 °C, 5% CO_2_ and 95% humidity in an incubator. The culture medium was changed every three days by centrifuging the suspended cells at 1300 rpm for 7 min and re-suspending the resulting pellet in fresh medium as previously described [1]. The BMMCs were used after 4 weeks in culture as described in Table 1.

**Table 1:** Outline of the different treatments administered prior to the transcriptomics study.

**Sample name**	**Genotype**	**Treatment**
PEP+/+ IgE	PEST-domain-enriched tyrosine phosphatase WT	Immunoglobulin E
PEP+/+ IgE DNP	PEST-domain-enriched tyrosine phosphatase WT	Immunoglobulin E + dinitrophenol
PEP+/+ IgE DEX	PEST-domain-enriched tyrosine phosphatase WT	Immunoglobulin E + dexamethsone
PEP+/+ IgE DNP DEX	PEST-domain-enriched tyrosine phosphatase WT	Immunoglobulin E + dinitrophenol + dexamethsone
PEP−/− IgE	PEST-domain-enriched tyrosine phosphatase mutant	Immunoglobulin E
PEP−/− IgE DNP	PEST-domain-enriched tyrosine phosphatase mutant	Immunoglobulin E + dinitrophenol
PEP−/− IgE DEX	PEST-domain-enriched tyrosine phosphatase mutant	Immunoglobulin E + dexamethsone
PEP−/− IgE DNP DEX	PEST-domain-enriched tyrosine phosphatase mutant	Immunoglobulin E + dinitrophenol + dexamethsone

### 2.2 Preparation of libraries, sequencing and data analysis

**Fig. 1:**
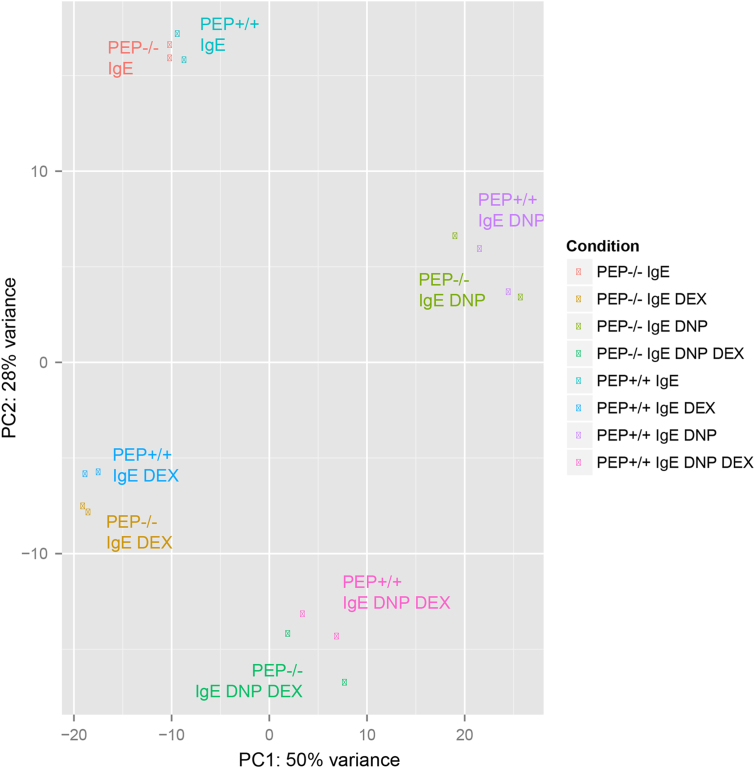
Principal Component Analysis (PCA) plot of normalized expression for the samples.

**Fig. 2:**
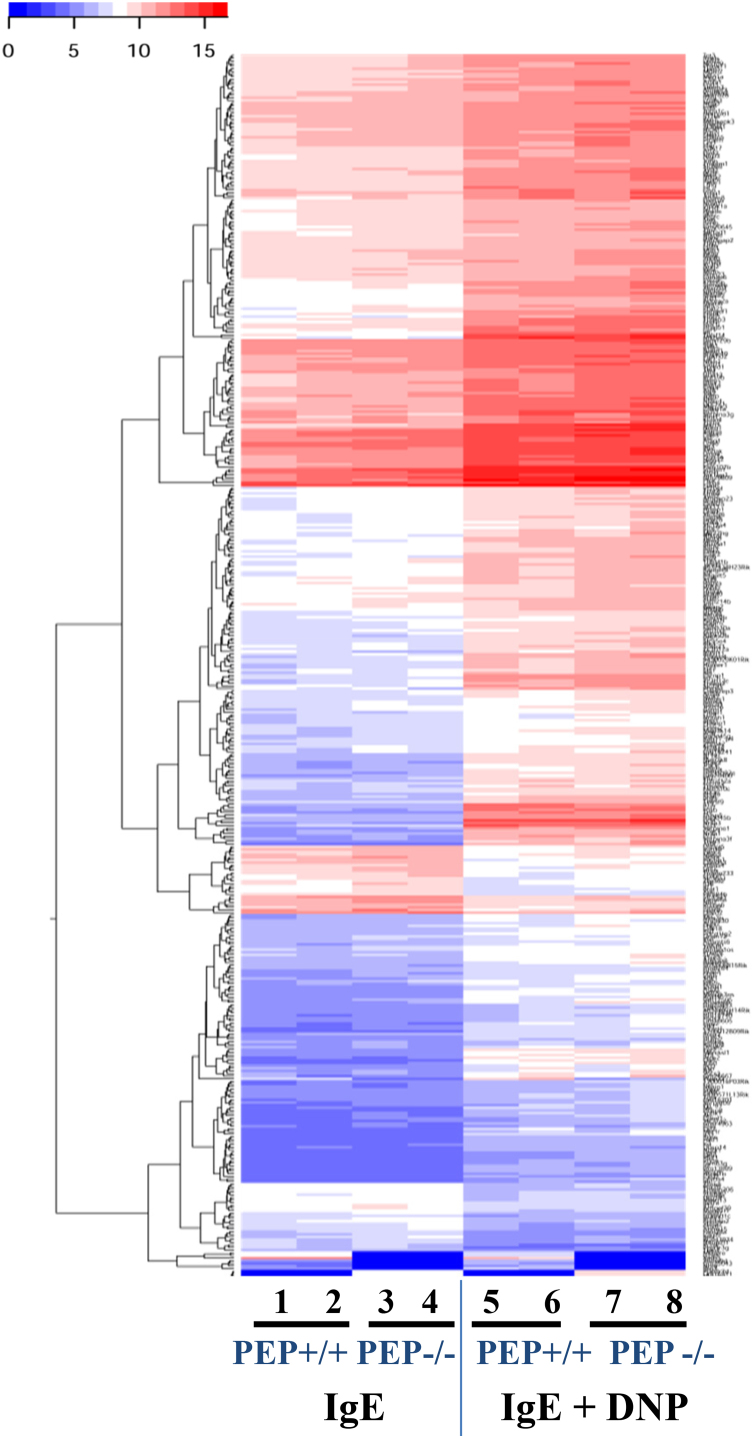
Heatmap illustration of gene expression in sensitized (IgE) and activated (IgE + DNP) mast cells: Color-coded heatmap of the normalized expression level of genes representing two replicates each for PEP+/+ and PEP−/− BMMCs. The color gradient shows blue for low and red for high expression of genes.

**Fig. 3:**
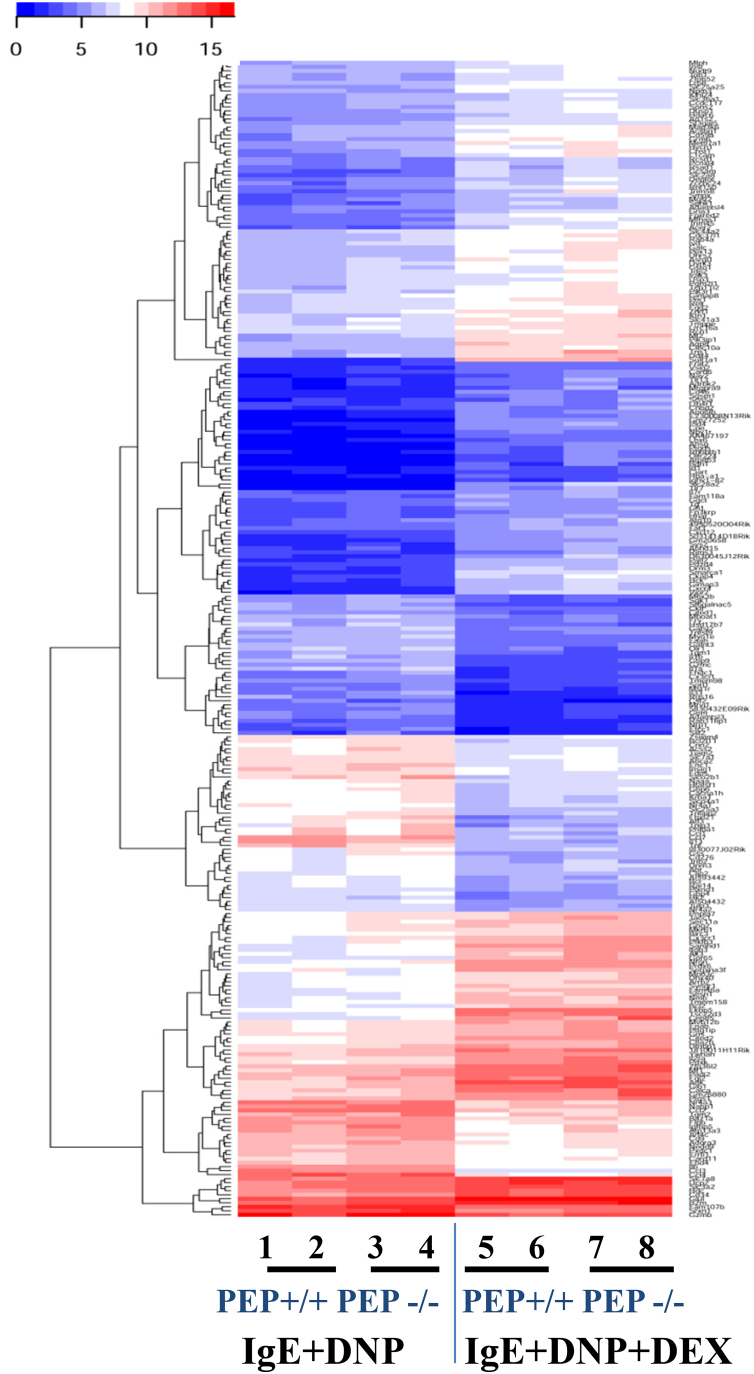
Heatmap illustration of gene expression pattern in activated (IgE + DNP) and activated mast cells treated with dexamethasone (IgE + DNP + DEX). Color-coded heatmap of the normalized expression level of genes representing two replicates for PEP+/+ and PEP−/− BMMC. The color gradient shows blue for low expression and red for high expression.

For the RNAseq experiment, total RNA was extracted from 3×10^6^ PEP+/+ and PEP−/− BMMCs using innuPREP RNA Mini Kit (Analytik Jena AG, Jena, Germany). A total of 1 µg RNA was used to prepare mRNA sequencing libraries for each sample using the TruSeq stranded mRNA kit v2 (Illumina), according to the vendor´s protocol. Next, 10 pM of multiplexed libraries were used to generate clusters in 2 lanes of a high-throughput flowcell. A HiSeq. 1500 was used to obtain paired-end reads of 50 bases using the sequencing by synthesis (SBS v3 kit Illumina). The cluster detection and the base calling were done with the software RTA (v1.13 Illumina) and demultiplexing with the software CASAVA (v1.8.1 Illumina). The sequencing resulted in 576 million reads with a mean quality Phred score of 35.2. The quality of the sequencing data was first assessed with FASTX toolkit (v0.0.13 [http://hannonlab.cshl.edu/fastx_toolkit/]) and no pre-processing was needed. The alignment of the sequencing reads was done with Tophat2 (v2.0.11) [2] against the mouse reference genome (GRCm38 v75). The raw count per gene was calculated using HTSeq (v0.5.3) [3]. The normalization of the counts, the differential expression analysis were done using the R software packages including DESeq. 2 [4]. The reproducibility of the biological replicates for all the conditions examined was assessed by Principal Component Analysis (Fig. 1). The significantly deregulated genes for each paired comparison were subjected to hierarchical clustering using *hclust* and *gplot* R packages. These were done for the activation of the mast cells by antigen (DNP) alone (Fig. 2) or together with DEX treatment (Fig. 3).

### 2.3 Validation of distinct target genes

**Fig. 4:**
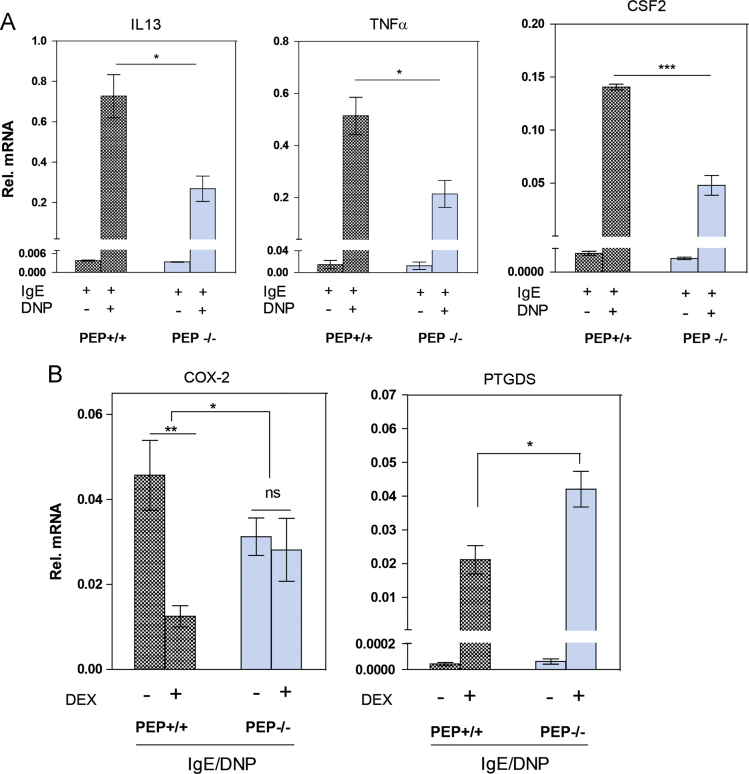
qRT-PCR validation of the expression of some genes differentially misregulated in the RNA-seq studies. **A**. Gene expression pattern of cytokine/chemokine genes identified to be differentially misregulated in response to antigen (IgE + DNP) compared to IgE treatment. The results are presented as the mean ± standard error of mean. * *p* < 0.05, *** *p* < 0.001 for unpaired two-tailed *t* test (*n* = 3). **B**. Dexamethasone-induced regulation of expression of lipid mediator genes, COX-2 (cyclooxygenase-2) and PTGDS (prostaglandin D2 synthase - lipocalin-type) identified as differentially misregulated in PEP−/− BMMCs compared to the PEP+/+ BMMCs. The results are presented as the mean ± standard error of mean. * *p* < 0.05, ** *p* < 0.01 for unpaired two tail *t* test (*n* = 5).

KEGG pathway analysis of genes significantly deregulated upon PEP gene deletion was carried out using DAVID [5]. Genes associated with antigen receptor (FcεRI signaling pathway) (IL 13, TNFα, and CSF2) and arachidonic acid metabolism pathway (COX-2, PTGDS) were some of the key genes identified to be deregulated in response to antigen and glucocorticoid respectively. A further validation of these results was carried out using qRT-PCR analyses. 1 µg of RNA was reversed transcribed to complementary DNA (cDNA) and used for real-time PCR (StepOnePlus Life Technologies, Carlsbad, California) with gene-specific primers for the following genes: IL 13 (Forward 5′-TGGCTCTTGCTTGCCTTGGT-3′, Reverse 5′-TTTTGGTATCGGGGAGGCTGG-3′), TNFα (Forward 5′-GATCGGTCCCCAAAGAAGGGATG-3′, Reverse 5′-TGATCTGAGTGTGAGGGTCTCG-3′), CSF2 (Forward 5′-TCGTCTCTAACGAGTTCTCCTT-3′, Reverse 5′-CGTAGACCCTGCTCGAATATCT-3′), COX-2 (Forward 5′-TGAGCAACTATTCCAAACCAGC-3′, Reverse 5′-GCACGTAGTCTTCGATCACTATC-3′), PTGDS (Forward 5′-GCTCCTTCTGCCCAGTTTTCCT-3′, Reverse 5′-GGAGGACCAAACCCATCCAC-3′) and in parallel with a reference gene Ribosomal protein large P0 subunit (Rplp0) (Forward 5′-GGACCCGAGAAGACCTCCTT-3′, Reverse 5′-GCACATCACTCAGAATTTCA-3′). The gene-specific primers were designed using the publicly available web-based tool ‘Primer-BLAST’ NCBI-NIH. The relative mRNA transcript abundance for the test genes in each sample was calculated using the 2^−(ΔΔCT)^ method [6] for the role of PEP in antigen-mediated mast cell activation (Fig. 4A) and the effect of DEX on antigen-mediated mast cell activation (Fig. 4B).
